# Conflict side of creativity: Role of supervisory support and team affective tone in facilitating creative idea validation

**DOI:** 10.3389/fpubh.2022.965278

**Published:** 2022-10-20

**Authors:** Ahmad Adeel, Daisy Mui Hung Kee, Yahya Qasim Daghriri

**Affiliations:** ^1^Department of Business Education, The University of Chenab, Lahore, Pakistan; ^2^School of Management, Universiti Sians Malaysia, Penang, Malaysia

**Keywords:** relationship conflicts, creative idea validation, supervisory support, team affective tone, belongingness, creativity, innovation

## Abstract

**Purpose:**

We seek to understand whether relationship conflicts of co-workers affect the validation of creative ideas or not. Furthermore, what boundary conditions may help prevent potential drawbacks of relationship conflicts with co-workers to validate their creative ideas?

**Design/methodology/approach:**

The proposed model was tested by using multisource data collected across two points in time from final year nursing students and medical dispensers of five nursing colleges of south-Punjab, Pakistan. The model was analyzed with Mplus for random coefficient models for direct effects, mediated moderation, and UCINET for central tendency of creative idea validation.

**Findings:**

It was found that relationship conflicts with co-workers were negatively related to their validation of creative ideas. However, supervisory support and team affective tone independently attenuate the negative effects of relationship conflicts with co-workers and the validation of creative ideas. Positive affective tone emerged as a positive predictor of creative idea validation. Additionally, positive affective tone as affected by supervisory support attenuated the negative relationship between relationship conflicts with co-workers and their validation of creative ideas. Finally, the relationship between relationship conflicts with co-workers and their validation of creative ideas is more positive when both supervisory support and positive affective tone are high, however, low otherwise.

**Practical implications:**

This study will help policymakers understand what might be hindering the transfer of creative ideas to influential others (Leaders, Managers, etc.) and what they need to do to enhance the creative pool of their organizations. Although developing an environment that fosters creativity is important for the organizations, developing strategies to manage relationship conflicts related to supervisory support and positive affective tone will help transfer creative ideas to higher offices even when there are dysfunctional conflicts.

**Originality/value:**

This research shifts the conventional focus of understanding creativity from the generating side by explaining challenges that creative individuals face in promoting creative ideas with more criticism and offense by coworkers than support. Also, the interplay between the relationship conflicts with co-workers and team affective tone affected by supervisory support for validation of creative ideas enhanced our understanding of the boundary conditions of relationship conflict and creative idea validation.

## Introduction

Innovation: the implementation of creative ideas, includes several stages, creativity: the generation of novel and useful ideas is the obvious point of departure for innovation to take place (1–4). However, another important stage in this process is ideas validation where the idea generator approach co-workers for refining their ideas, in social interactions (3, 5), before any formal approval of the competent authorities (5–7). Validated ideas adds to the idea pool of the organization (8), however, ideas which fail to find any validation or endorsement just increase sunk cost for the organizations as they fail to contribute to their organization (2, 9). Given that idea validation is critical for the organizations (3, 10), identifying its antecedents has become a pressing issue in contemporary research (1, 3, 11, 12).

Relationship conflicts refer to the tension stemming from interpersonal incompatibilities in personalities and emotions (13) are part of organizational life (14). These conflicts, are detrimental to creative process (3, 6, 10, 15, 16). Thereby, researchers have highlighted the importance of studying relationship conflicts so as to ensure that employees work properly (17) and organizations can capitalize on the creative potential of their employees (3, 18, 19). Whereas, idea validation is predominantly inflected by social interactions and influence (1, 3); Social conflicts on the part of employees is detrimental to organizations because it may inhibit the organization to capitalize on employees' creativity (20, 21). Thereby, investigating creative idea validation in the presence of relationships conflicts is significant for the research. More specifically, it is critical to understand both when co-workers validate ideas of those with whom they maintain relationship conflicts and how the negative effects of relationship conflicts can be reduced for validation of creative ideas. Our research further contributes to filling this gap.

We build on the belongingness theory (22) that explains the fundamental role of interpersonal relationships and the distal consequences (22, 23). The overarching tenant of the theory is that positive relationships are translated into supportive behaviors; however, dysfunctional relationships avoid interactions with the conflicts' targets. Thus, the belongingness theory is distinctively placed in both resolving and explaining this complication in the creative process. By following the contingency perspective (19, 24), based on previous findings that supervisory support may moderate the extent to which conflicts may have beneficial consequences (25, 26). Also, according to the contingency perspective on the conflict-outcome relationship, team affective tone as collectively interactive activities (24, 27) may serve as boundary conditions to explain the extent to which interpersonal conflicts may bring beneficial outcomes. Thus, we propose that when supported by supervisors for a positive team affective tone, a conflict-provoking person will get validation of his creative ideas, thereby buffering the negative influence of relationship conflicts on creative idea validation.

The current study contributes to the literature in several ways. First, employee creativity research has predominantly focused on the idea generation part of creativity, ignoring the challenges creative individuals face in promoting creative ideas with more criticism and offense by coworkers than support (1, 28, 29). Additionally, communication between coworkers plays a significant role in the creative process (15, 19, 30). Less is known about the role and impact of communication hazards for creative idea validation. Second, using contingency perspective, we identify interplays between the relationship conflicts with co-workers and team affective tone as affected by supervisory support for validation of creative ideas that may clarify that under what conditions detrimental effects of conflicts can have repercussions for focal employees' creativity. The study addresses the importance of a supportive environment, including supervisory support and affective team tone in creating buffer between negative consequences of relationship conflicts between co-workers.

Third, communication between coworkers plays a significant role in their creative idea validation of their colleagues. In this study, we have not only examined the effects of conflict on creative idea validation but also identified the importance of the dual process of communication (including verbal and non-verbal cues) in creating buffering effects in explaining the reduction of harmful effects of relationship conflict on creative idea validation by co-workers. Finally, in the creative process, the empirical research predominantly focused on variance-focused creativity (problem identification, information searching, idea generation); little is known about selection-focused creativity (idea validation, idea endorsement) (3, 31). Overall, due to the practical value of how people receive creativity, the need to understand the receiving side of creativity has been raised (32, 33). Thus, with this research, we have made some distinct contributions to creativity and conflict research.

## Literature review and hypothesis development

### Relationship conflicts and creative idea validation

The belongingness theory (22) establishes and explains the fundamental role of interpersonal relationships in explaining human lives and behaviors. According to this theory, predominantly individuals have a strong need to belong; thus, they seek interpersonal contacts and cultivate desired relationships. In an ideal situation, these interpersonal contacts are free from conflicts and negative effects, positive in nature, and produce affectively pleasant behaviors. However, based on the nature of interpersonal relationships, interpersonal relationships have their distal and proximal consequences. Broadly, as explained by the theory (22), when individuals have dysfunctional relationships with others, they may experience being avoided by others and may fail to obtain required feedback and resources (22). Thus, in our research, belongingness theory is distinctively placed for explaining the role of relationship conflicts of creative individuals and validation of creative ideas by peers at work.

Co-workers' relationship and its impact on co-worker creative idea validation are important issues that need attention in the literature. Although creative idea generation is a solitary activity (32, 34), however, relationship contexts in a workgroup impact an individual's actions. Employees' relationship with their co-workers may impact the degree to which they are motivated to get engaged with creative undertakings (35–39). In a workgroup environment, employees interact with their co-workers the way they interact with their supervisors. These interactions include both work and non-work activities/tasks, which can impact their behavior generally and their creative performance particularly as it is the consequence of these behaviors (34, 36, 37, 40). Literature has identified that not only group characteristics including size, gender profile work, the experience of members, etc. but, group dynamics in the form of cohesion, interaction and communication process between its members also have a profound influence on its member's creative performance (37, 41, 42).

Previous research on co-worker relationships (43, 44) acknowledges that communication about ideas occurs during all stages of the creative process. During earlier stages of the creative process, individuals share their knowledge with their co-workers and receive input from them. This input can be related to relevant task knowledge or complete change in perspectives (45). These co-workers' interactions might revamp ideas and be considered a foundation for idea incubation (5). So, when there are relationship conflicts between co-workers, there will be limited communication between them (22, 46). This lack of relationship or relationship conflict between co-workers would negatively affect creative idea validation. Based on the above discussion following hypothesis is placed

**H1: Relationship conflicts with co-workers are negatively related to their validation of creative ideas**.

### Moderating impact of supervisory support

Supervisory support is linked to a higher level of subordinate creativity (47, 48). Literature also supports the link between the values of supervisors and organization innovation rates (49). We have to test whether the supervisory behavior/support has a moderating impact on the antagonistic relationship between co-workers and their creative idea validation. Supervisors influence subordinates through various forms, including role modeling, goal definition, reward allocation, resource distribution, communication of organizational norms and values, structuring of workgroup interactions, conditioning subordinates' perceptions of the work environment, and influence over processes and procedures used (50–52), ultimately influences employee creativity (53, 54). Similarly, employees' perceptions regarding autonomy, support, trust, and goal clarity contribute to creative idea generation (55, 56) and innovation (57).

Supervisory support also has a psychological influence on employees (58–60) that influence their feelings to develop positive feelings in subordinates through self-efficacy. Employees' feelings influence their work (61), and supervisory support help to create positive feelings in subordinates through self-efficacy. These psychological states result in two outcomes, first, the effectiveness and second, the innovative behavior of subordinates (60, 62). Thus, we propose that supervisory support acts as a buffer in the negative relationship of relationship conflict and coworkers' creative idea validation. The psychological empowerment in subordinates due to supervisory support not only induces creativity but also helps in making the employees feel that they are secure as they have support from their supervisors. Similarly, when co-workers are clear that one particular employee has support from the supervisor, they are less likely to reject the creative idea of that employee regardless of whether they have relationship conflict with that employee. Hence, based on above discussion following hypothesis is proposed

**H2a: Supervisory support attenuates the negative effects of relationship conflicts with co-workers on their validation of creative ideas, such that relationship conflict's negative impact on co-workers' creative idea validation is even less when supervisory support is high**.

### Moderating impact of team affective tone

Team affective tone is defined as “consistent or homogeneous affective reactions within a group” (63). These affective reactions are shared perceptions of moods and emotional states of team members (64) and can be considered the aggregate moods of team members (65). The shared emotions at the team level can be demonstrated as “team affect.” The prior literature has been proposed that team dynamics, effectiveness, and creativity is influenced by team affective tone (66–68). The positive affective moods and behaviors are linked with performance, creativity, and coordination of team members (65, 68, 69). The emotional contagion process through which the state of one team member is transferred to another team member is one of the significant causes of team affective tone (65, 70).

The team members' creative processes and information processing are the two theoretical mechanisms behind the link between the team's affective tone and the creativity of its members (68). The working environment in which the team members operate has an impact on the willingness of the team members to work together and engage in creative work solutions (71, 72). When there are enjoyable interactions with team members, they are more likely to share and discuss their ideas and develop better and creative answers (68, 73). A positive team affective tone works by facilitating team members' creative processes. These positive work interactions help enhance information processing by allowing team members to access additional information through ideas exchanges during group discussions (74). A positive team affective tone develops a working environment where employees set aside their relationship conflicts and achieve collective goals. Team affective tone reduces the harmful effects of relationship conflicts on the coworker's creative idea validation and acts as a buffer. Based on the above discussion following hypothesis is proposed

**H2b: Team affective tone attenuates the negative effects of relationship conflicts with co-workers on their validation of creative ideas, such that, relationship conflict's negative impact on co-workers' creative idea validation is even less when team identification and co-operation are high**.

Our preceding hypothesis proposes that supervisory support produces a positive team affective tone; a positive team affective tone acts as a buffer in transmitting the negative impact of relationship conflict on coworker's creative idea validation. It is predicted that a positive team affective tone mediates the moderating effect of supervisory support on the relationship between relationship conflict and coworkers' creative idea validation (Hypothesis 1), constituting a case of mediated moderation (75). Although mediated moderation can take multiple forms, the type of mediated moderation that we expect is present when (1) a variable (supervisory support) moderates the relationship between an independent variable (relationship conflict between coworkers) and a dependent variable (coworker creative idea validation), as in Hypothesis 1; (2) the moderating variable (supervisory support) causes a mediating variable (positive team affective tone); and (3) the mediating variable (positive team affective tone) moderates the relationship between an independent variable (relationship conflict between coworkers) and a dependent variable (coworker creative idea validation), thereby transmitting—and eliminating—the moderating effect of the original moderator (supervisory support). Having already proposed these relationships, we present a hypothesis for mediated moderation: supervisory support attenuates the negative effects of relationship conflicts with co-workers on their validation of creative ideas through a positive team affective tone.

**H2c: Team affective tone mediates the moderating effect of supervisory support on the relationship between relationship conflicts with co-workers and creative idea validation**.

“Verbal and non-verbal behavior produced with the intention of providing assistance to others perceived as needing that aid” (76). A growing body of literature has sought to understand the connection between social support and positive outcomes in individuals by looking at the dual process of supportive communication. The Dual-process of communication identifies the importance of both verbal and non-verbal communication, i.e., content and perception about the message. We are proposing here that the dual process of communication plays its role when there is relationship conflict, and coworkers do not validate the creative ideas of their colleagues. In this dual-process of communication, the verbal cues coming from the supervisory support, and non-verbal cues come from the team affective tone. We propose here that both the verbal and non-verbal cues act as a buffer and moderate the negative effects of relationship conflict on creative idea validation by coworkers. Supervisory support and a positive team affective tone jointly reduce the gap between coworkers in relationship conflict.

**H3: Supervisory support and team affective tone will jointly moderate the negative effects of relationship conflicts with co-workers on co-workers' validation of creative ideas, such that, relationship conflicts' negative impact on co-workers' validation of creative ideas is even less when supervisory support and team affective tone are high**.

**Figure 1:**
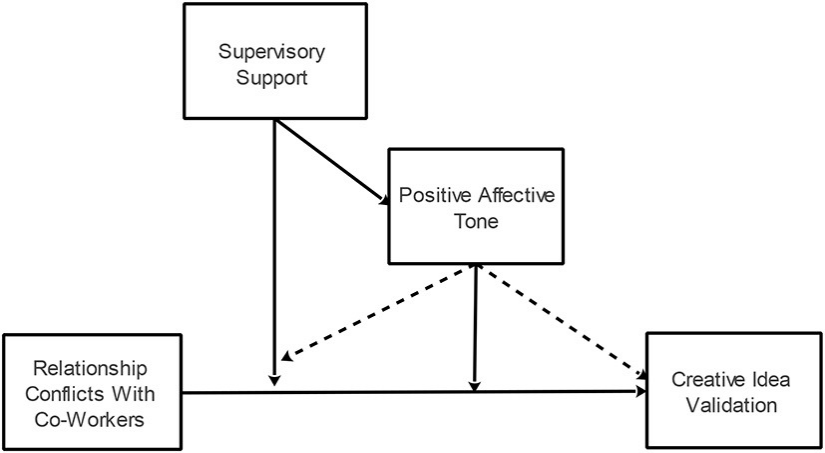
Research model.

Our research model is presented in Figure 1.

## Methodology

### Participants

The characteristics of the participants of this study provided an opportunity to test the proposed model in the health care sector of Pakistan. Previous researchers have used non-probability sampling by recruiting a variety of adequate participants (14, 77–79) for generalizability of their research findings. Therefore, follow owing these examples, in this research; we approached five public sector medical colleges of south-Punjab for data collection, to initiate the process of convenience sampling.

For sample selection, statistical power, significance level, effect size and the number of independent variables are important to take into account (80). When maximum number of independent variable in a model are two, in order to achieve a statistical power of 80% for detecting *R*^2^ values of at least 0.1 with a significance level of 5%, at least participants needed are 90 (81, 82). In our study, data collection process was initiated with 578 participants, which is larger than the minimum sample size of 90 participants.

Common method variance is an issue related to survey studies, however, the temporal separation of data collection of study variables, reduce evaluation oppression, and protecting respondent anonymity can reduce the chances of common method variance issues (83). As a procedural technique, following previous research, we divided data collection process into two points in time, we maintained respondent anonymity by allocating dummy codes to the respondents, and reduced evaluation apprehension by sending direct email to the respondents so that they may respond at a time and place of their convenience (84). Additionally we also performed Harman's single factor test, the value for percentage of variance was 24.735% of the total variance which indicates that common method biases were not a serious problem (85).

### Sample and data collection

All enrolled final year generic nursing students and medical dispensers of public sector nursing colleges (Nishtar Medical College, Multan; B.V. Hospital, Bahawalpur; DHQ Hospital, Layyah; DHQ Hospital, D.G.Khan; DHQ Hospital, Mianwali) of south-Punjab, Pakistan (*N* = 578). Initially, in a formal meeting, the purpose and significance of the study were discussed with relevant District Health Officers (DHO) and Medical Supertendents. The data collection process was divided into two points in time with the multisource collection technique. To identify the individual response with leaders' and co-workers' responses, we assigned dummy codes to all the respondents. In introductory seminars at all of the hospitals, we introduced our research and then the HR officer sent an introductory email to all 578 respondents and their 47 relevant supervisors; we then initiated our data collection process. We emailed employees for their responses about relationship conflicts with co-workers and supervisory support (Time 1). This technique of data collection also ensured the safety of the participants at the time of covid-19 pandemic when social distancing was a requirement. We received an initial response from 497 respondents, and then we initiated the second phase of data collection, which was more like a social network analysis. After 4 weeks, we sent questionnaires to those 497 respondents who had already provided their responses at time 1 (Time 2). The response was received from 431 respondents about creative idea validation and team affective tone. Data for all of the control variables were also obtained at the time 1.

For the final data set of this study, we focused on the matched data of employees and their co-workers. The mismatched and incomplete response was not included in the final data set which yielded a response of 243. Due to the complex nature of response required at time 2, the final data set dropped from 497 to 243. A final qualified sample of 243 was used in all of this study's analyses and model testing. In the final qualified sample, 34.5 were males, and 65.5 were females; the average experience working in the healthcare sector was 5 years. We used the maximum likelihood method for missing values, which is a more robust technique compared to list-wise deletion, pair wise deletion, mean replacement, or multiple imputation methods (86–88).

### Measures

#### Relationship conflict with co-workers

Relationship conflict with co-workers was measured on a self-reporting measure of three items-five points Likert type scale (46, 89). The three items of the scale were “How strong is your personal- conflict with your co-workers?”; “How strong is your personality-based conflict with your co-workers”; and “How strong is your personal friction with your co-workers?.” Scale items range from 1 = “Strongly disagree” to 5 = “Strongly agree.” (α = 0.86).

#### Idea validation

Coworker's rated idea validation was measured as the number of co-workers with whom focal employee interacts as a part of their job with five items-five point Likert type scale (3). Egocentric network technique was used using name generation and interpreter method. As the first step of this method, for name generation, the employees were asked to recall and list down names of the co-workers of their choice based on the criteria: a) with whom they have to interact for task-related activities; b) whose feedback and support is essential to complete work, and c) who are dependent upon them to complete their task. In the interpreter step, we asked the respondents to provide data for co-workers on their list for their creative idea validation. To mitigate any social concern, we did not restrict them to rate every member of their work unit. Sample items for the scale are “I provide my opinion to the focal employee about his/her new ideas.,” “I provide feedback to the focal employee about the feasibility of his/her new ideas.”, and “I talk to the focal employee about his ideas to see if they will work.” To calculate creative idea validation for a focal employee, consistent with previous research, we used UCINET 6.347 (90), which measures central tendency (91).

#### Supervisory support

Supervisory support was measured with four items-five point Likert type scale (50, 92). Sample items include “How true is it that your supervisor is warm and friendly when you have problems?” and “How true is it that your supervisor shows approval when you have done well?” Scale items range from 1 = “Strongly disagree” to 5 = “Strongly agree” (α = 0.89).

#### Positive affective tone

Positive affective tone was measured with PANAS scales (71, 93) with five items-five point Likert type scale. As our focus in this research was positive team affective tone, therefore, consistent with previous research, we used words such as excited, enthusiastic, and inspired (94). We asked the respondents about their feelings when they think or talk about their work team (α = 0.79).

#### Control variables

In this study, we controlled for demographic and contextual variables that may affect and provide alternative explanations of creative idea validation for the focal employee. We controlled for gender and professional experience with one question each. We further controlled for psychological safety with seven items-five point Likert type scale (95) (α = 0.93), autonomy with four items-five point Likert type scale (96) (α = 0.87), extrinsic motivation with twelve items-five point Likert type scale (97) (α = 0.78), and intrinsic motivation with four items-five point Likert type scale (98) (α = 0.84).

### Analytical strategy

Mplus 7.0 was used in all of the analyses of this study. We collected data from generic nursing students who were nested into other teams under different supervisors based on their assigned healthcare assignments. In situations like this, the use of simple regression techniques could underestimate the standard error; additionally, there could be potential interdependence among the study variables (99). Scholars recommended using random coefficients analysis techniques (100). In our sample, all variables were operated at a single level of analyses; thus, we used the random coefficients modeling technique at the individual level with Mplus 7.0 for random coefficients. Researchers have already used this technique for data with similar characteristics (15, 77). For model fit indicators, the output produced by Mplus cannot be used in a regular way; therefore we also have to perform the Satorra-Bentler difference test using the log-likelihood method for chi-square difference testing (101). Before any analysis, we grand mean centered all the variables of this study; additionally, to reduce chances of multicollinearity for interaction variables, we also grand mean centered interaction variables (102).

### Data analyses

## Results

Descriptive statistics and zero-order correlation among study variables are shown in Table 1. Although, results of Satorra-Bentler difference test using log-likelihood method for chi-square difference test performed for model fit indicators are presented in Table 2, conventional model fit indicators for the final model are also provided. The conventional statistics for final model, Chi-square baseline model χ^2^ = 58.979, 13, *p* < 0.001, loglikelihood for alternate model = −296.991, with scaling correction factor 1.104, loglikelihood for null model = −322.53, with scaling correction factor 2.017, CFI = 0.99, TLI 0.99, error variance for null model = 0.064, error variance for alternate model = 0.049, and RMSEA = 0.0001 with construct reliability of 0.83 for average variance extracted (AVE) indicated a good fit of the model to the data.

**Table 1:** Means, standard deviation, and correlation among study variables.

**Variable**	**Mean**	**SD**	**1**	**2**	**3**	**4**	**5**	**6**	**7**	**8**	**9**
1. Gender	0.66	0.48									
2. Professional experience	6.09	2.10	0.045								
3. Psychological safety	3.74	0.85	−0.025	0.026							
4. Autonomy	3.40	0.97	−0.013	0.012	0.474^**^						
5. Extrinsic motivation	3.91	1.47	−0.130^*^	−0.106	−0.201^**^	−0.072					
6. Intrinsic motivation	3.70	1.31	0.008	0.067	0.179^**^	0.128^*^	0.039				
7. Relationship conflict with co–workers	3.77	1.59	0.049	0.075	0.094	0.007	0.032	0.279^**^			
8. Supervisory support	3.86	1.52	0.059	0.110	0.127^*^	−0.007	0.027	0.087^**^	0.196^**^		
9. Positive affective tone	3.69	0.86	0.012	0.149^*^	−0.023	−0.159^**^	0.067	0.123^**^	0.478^**^	0.089^**^	
10.Creative idea validation	3.80	0.75	−0.054	−0.090	−0.195^**^	−0.153^**^	0.109	−0.103	−0.137^*^	−0.086	0.099

Observations = 243. Clusters = 41. Gender was coded as 0 = Female, 1 = Male. Professional Experience was measured in years.

* p < 0.10.

** p < 0.05.

^***^p < 0.01.

**Table 2:** Random coefficients regression analyses.

**Predictor**	**Model 1**		**Model 2**		**Model 3**		**Model 4**		**Model 5**		**Model 6**	
	**Creative idea validation**		**Creative idea validation**		**Positive affective tone**		**Creative idea validation**		**Creative idea validation**		**Creative idea validation**	
	**Estimate**	**SE**	**Estimate**	**SE**	**Estimate**	**SE**	**Estimate**	**SE**	**Estimate**	**SE**	**Estimate**	**SE**
Gender	−0.056	0.080	−0.053	0.046	−0.046	0.043	−0.062	0.082	−0.037	0.081	−0.036	0.080
Professional experience	−0.024	0.022	0.032^*^	0.013	0.038^**^	0.013	−0.023	0.023	−0.035	0.022	−0.035	0.022
Psychological safety	−0.122	0.077	−0.061	0.044	−0.007	0.045	−0.092	0.080	−0.092	0.077	−0.111	0.081
Autonomy	−0.080	0.053	−0.133^***^	0.035	−0.110^***^	0.031	−0.047	0.052	−0.012	0.053	−0.006	0.055
Extrinsic motivation	0.032	0.033	0.014	0.027	0.007	0.025	0.027	0.029	0.026	0.026	0.029	0.026
Intrinsic motivation	0.105	0.080	0.128	0.097	0.148	0.092	0.062	0.079	0.005	0.075	−0.011	0.080
Relationship conflict with co–workers	−0.131^*^	**0.063**	0.048	0.090	0.258^***^	0.081	−0.044	0.107	−0.317	0.216	−0.627	0.563
Supervisory support			0.300^***^	**0.077**	0.440^***^	**0.078**	0.230^***^	0.078	−0.058	0.167	−0.249	0.390
Relationship conflict with co–workers x supervisory support					0.047^***^	0.007	0.035^*^	**0.018**	0.012	0.038	0.167	0.108
Positive affective tone									0.429^*^	0.216	0.417	0.362
Relationship conflict with co–workers x positive affective tone									0.096^**^	**0.040**	0.079	0.129
Supervisory support X positive affective tone											0.142	0.122
Relationship conflict with co–workers X Supervisory support X positive affective tone											0.467^**^	**0.062**
Δ χ 2 (Δdf)	3619.97 (21)		3745.84(20)		3230.02(19)		3412.74(19)		2161.16(17)		58.32(12)	
Δ *R^2^*	0.171		0.187		0.546		0.187		0.250		0.234	

Observations = 243. Clusters = 41. Gender was coded as 0 = Female, 1 = Male. Professional Experience was measured in years.

Δ χ 2 refers to Satorra–Bentler scaled chi–square difference test Muthén and Muthén (1998–2010). Δdf is change in degree of freedom.

Δ R^2^ is degree of reduction in error variance.

* p < 0.10.

** p < 0.05.

*** p < 0.01.

Bold value indicates significant and important values model.

### Test of hypothesis

Using Mplus 7.0 for the random coefficient model; we regressed gender, professional experience, psychological safety, autonomy, extrinsic motivation, intrinsic motivation as control variables along with the relationship conflicts with co-workers as an independent variable on creative idea validation to confirm the direct effect of relationship conflicts with co-workers on their validation of creative ideas. The results of this analysis are presented in table2-model1, the significant coefficient **(β** **=** **−0.131**, ***p* ≤** **0.05)** confirmed the negative effects of relationship conflicts with co-workers on creative idea validation, thereby providing support to hypothesis 1 of this study. Although not hypothesized, in table2-model2, we regressed gender, professional experience, psychological safety, autonomy, and extrinsic motivation, intrinsic motivation as control variables, and relationship conflicts with co-workers and supervisory support to confirm the direct effects of supervisory support on creative idea validation. The significant coefficient **(β** **=** **0.300**, ***p* ≤** **0.001)** confirmed the positive impact of supervisory support on the validation of creative ideas among co-workers.

We followed a three-step procedure for moderation analysis (102) and a three-step procedure for mediation (103). The indirect effect option could not be considered for our models as the bootstrap option cannot be used with random coefficient analyses (101). We regressed gender, professional experience, psychological safety, autonomy, extrinsic motivation, and intrinsic motivation as control variables, relationship conflicts with co-workers, supervisory support, and interaction of relationship conflicts with co-workers and supervisory support on a positive affective tone. We found a significant coefficient **(β** **=** **0.440**, ***p* ≤** **0.001)** of supervisory support and a significant coefficient **(β** **=** **0.047**, ***p* ≤** **0.001)** of the interaction term representing relationship conflicts with co-workers and supervisory support, which are presented in table2-model3.

**Figure 2:**
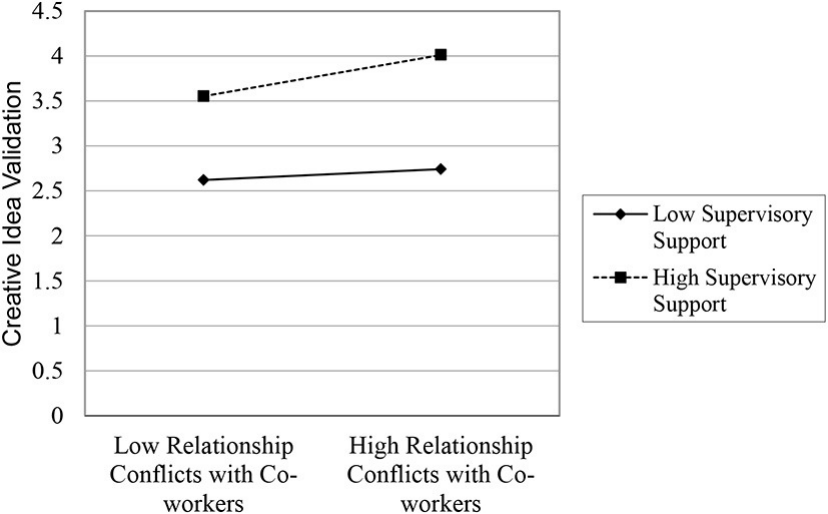
Plot of interaction between relationship conflict with co-workers and supervisory support.

We then regressed gender, professional experience, psychological safety, autonomy, extrinsic motivation, and intrinsic motivation as control variables, relationship conflicts with co-workers, supervisory support, and interaction of relationship conflicts with co-workers and supervisory support on creative idea validation. Significant coefficient confirmed the moderation of supervisory support **(β** **=** **0.035**, ***p* ≤** **0.001)** on the relationship between relationship conflicts with co-workers on their creative idea validation. The moderation of supervisory support attenuates the negative effects of relationship conflicts with co-workers on their validation of creative ideas. The moderating effects are presented in table2-model4 and shown in Figure 2, the plot of interaction suggested that high supervisory support will have a high positive impact on the relationship between relationship conflicts with co-workers and their creative idea validation. Even low supervisory support will positively affect the relationship between relationship conflicts with co-workers and their creative idea validation.

**Figure 3:**
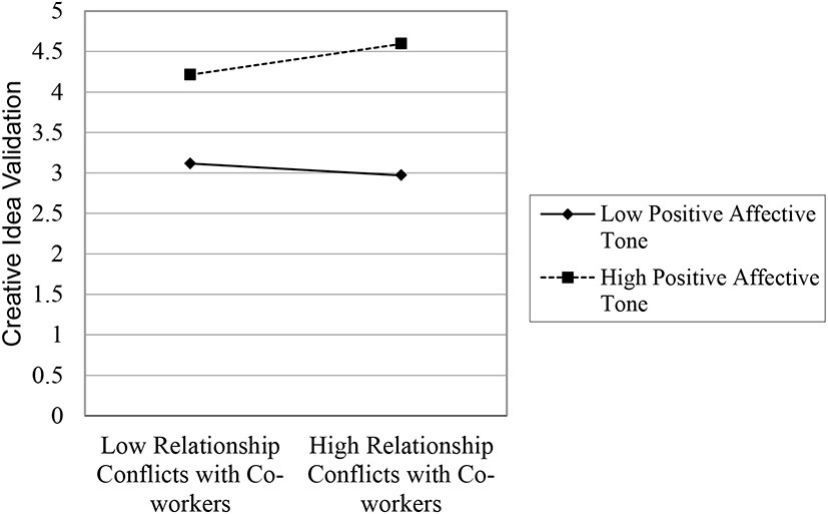
Plot of interaction between relationship conflict with co-workers and positive affective tone.

We then regressed gender, professional experience, psychological safety, autonomy, extrinsic motivation, intrinsic motivation as control variables along with relationship conflicts with co-workers, supervisory support, the interaction of relationship conflicts with co-workers and supervisory support, positive affective tone, and interaction of relationship conflicts with co-workers and positive affective tone on creative idea validation. In the presence of supervisory support and the interaction of relationship conflicts with co-workers and supervisory support, the interaction term of the relationship conflicts with co-workers and positive affective tone confirmed the moderating effect of positive affective tone **(β** **=** **0.096**, ***p* ≤** **0.01)**. The moderation of positive affective tone attenuates the negative effects of relationship conflicts with co-workers on their validation of creative ideas. The moderating effects are presented in table2-model5 and shown in Figure 3; the plot of interaction suggested that high positive affective tone positively; however, low positive affective tone negatively moderates the relationship between relationship conflicts with co-workers and their creative idea validation.

**Figure 4:**
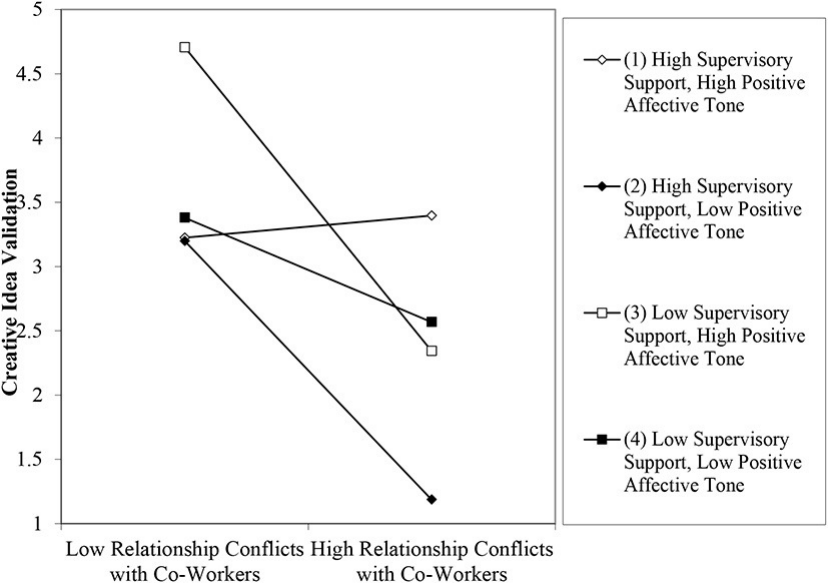
Plot of interaction between relationship conflicts with co-workers, supervisory support, and positive affective tone.

Finally, we tested a three-way interaction of the relationship conflicts with co-workers, supervisory support, and positive affective tone for its effects on co-workers' creative idea validation. We regressed gender, professional experience, psychological safety, autonomy, extrinsic motivation, intrinsic motivation as control variables along with relationship conflicts with co-workers, supervisory support, the interaction of relationship conflicts with co-workers and supervisory support, positive affective tone, and interaction of relationship conflicts with co-workers and positive affective tone, the interaction of supervisory support and positive affective tone, and a three-way interaction term of the relationship conflicts with co-workers, supervisory support, and positive affective tone on creative idea validation. Significant coefficient confirmed the effects of three-way interaction term on co-workers' validation of creative ideas **(β** **=** **0.467**, ***p* ≤** **0.05)**. The results are presented in table2-model6 and shown in Figure 4. The plot of three-way interaction suggested that high supervisory support and high positive affective tone will positively affect the relationship between relationship conflicts with co-workers and their validation of creative ideas, negative otherwise. We then confirmed the pattern of the results by slope difference tests (104). The results confirmed that high supervisory support and high positive affective tone slope was more positively significant (*t* = 2.93, *p* < 0.001) than high supervisory support and low positive affective tone (*t* = 2.32, *p* < 0.05), low supervisory support and high positive affective tone (*t* = 2.13, *p* < 0.05), and low supervisory support and low positive affective tone (*t* = 2.06, *p* < 0.05). This three-way interaction provided a clearer and accurate picture that even in the presence of relationship conflicts among co-workers, creative individuals will get their ideas validated by their co-workers when there is high supervisory support and high positive affective among them co-workers at work. The results can also be interpreted in another way that high supervisory support will create an environment of positive affective tone among co-workers that increases their validation of creative ideas even when they have relationship conflicts among them. Empirical findings of this study support all predictions of this study.

## Discussion

The main goal of this research was to uncover the possible effects of relationship conflicts among co-workers and their validation of creative ideas. First, negative effects of relationship conflicts on validation of creative ideas was found, when creative individuals are in relationship conflicts with others they are less likely to get their ideas validated by their co-workers. Second, support from the supervisors attenuates this negative relationship such that supportive supervisors foster an environment of positive affective tone in their work units which is beneficial for validation of creative ideas even in presence of relationship conflicts among co-workers.

Our results can be summarized as follows; first, we found that relationship conflicts with co-workers are negatively related to their validation of creative ideas. Second, supervisory support attenuates the negative effects of relationship conflicts with co-workers with their validation of creative ideas. The relationship is positive in the case of high supervisory support. Third, we found that positive affective tone attenuates the negative effects of relationship conflicts with co-workers with their validation of creative ideas. The relationship is positive in case of high positive affective tone; however, negative otherwise. Fourth, positive affective tone emerged as a positive predictor of creative idea validation. Fifth, positive affective tone affected by supervisory support attenuated the negative relationship between relationship conflicts with co-workers and their validation of creative ideas. Finally, the relationship between relationship conflicts with co-workers and their validation of creative ideas are more positive when both supervisory support and positive affective tone are high, however, low otherwise.

### Research contributions

#### Practical contributions

Employees can be creative in all functional areas of their jobs (16). Although creativity research has grown exponentially in enhancing our knowledge about the creative process (16), this knowledge largely rests on the theoretical foundations (105). Increasing demand to understand why the pace of innovation is still slow at organizations (106, 107) and does theoretically established concepts relate to actual performance (108). Thus, understanding the conflict side of creativity has practical implications. This study will help policymakers understand what might be hindering the transfer of creative ideas to influential others (Leaders, Managers, etc.) and what they need to do to enhance the creative pool of their organizations.

Although task conflicts have been found to support creativity, proper management of relationship conflicts will also benefit the creative process. Thus, developing strategies of relationship conflict management will also increase the creative potential of the organizations. Specifically, developing corporate-level strategies for supervisory support and team affective tone will increase the likelihood of co-workers' recognition and validation of conflict-provoking creative individuals' ideas for the benefit of organizations. The contingency perspective of this research also brings some valuable practical contributions. Although developing an environment that fosters creativity is vital for organizations, they also need to develop strategies to manage relationship conflicts among co-workers due to the creative environment. Therefore, we also recommend, organizations consider a relationship perspective when developing an environment for creativity: an environment based on mutual trust and respect so that a positive affective tone can establish with the support of the supervisors for the proper transferring of creative ideas to higher offices.

### Theoretical contributions

Building on belongingness theory (22), we have made some distinct contributions to the literature with this research. First, the primary contribution of this research lies in answering the fundamental question of how social conflicts of a conflict-provoking creative individual are related to the validation of ideas by peers. We built our conceptual model on belongingness theory. We uniquely integrated the contingency perspective of conflict literature from the lens of a supportive environment to answer how the odds of social conflicts with co-workers can be reduced in the creative process. Consistent with previous findings, we also found that odds of conflicts can be reduced if appropriately managed: the support from the supervisor and team members will attenuate the negative effects of conflicts on creative output (27, 109, 110). Additionally, previous research has established the role of team task conflicts and relationship conflicts in the creativity process (111). Empirical research has given little attention to understand the role of individual-level interpersonal conflicts in the innovative process (28, 112). To the best of our knowledge, the current study is the first empirical investigation of the relationship conflicts with co-workers and, as a reaction, its impact on their validation of creative ideas of that conflict-provoking creative individual.

Coworkers' presence and behavior matter in the creativity process (28). Interpersonal support and antagonism of co-workers subsequently influence the individual employee outcomes (113). Research has established that co-worker behaviors, support, and antagonism shape the social work environment for an individual. Organizations have moved from routine individual tasks to more complex and collective tasks (114), where work is mainly done based on interpersonal relationships (115) for goal achievements (116). But the reality is, interpersonal conflict is rife in modern organizations; employees have to work in organizations where they have to face more conflicts than supportive behaviors (117) (Psychometrics, 2009). Thus, investigating relationship conflicts as dysfunctional interpersonal interactions of co-workers was essential to examine for their validation of creative ideas.

The dual-process communication between coworkers plays a significant role in the creative idea validation of their colleagues. In this study, we have examined the effects of conflict on creative idea validation and identify the importance of the dual process of communication, including (verbal and non-verbal cues). We have extended the literature by empirically identifying the buffering effect of supervisory support and positive team affective tone acting as verbal and non-verbal cues when colleagues have to validate the creative ideas of those with whom they conflict. This research offers a more comprehensive understanding of the mechanism behind the creative idea validation in horizontal relationships in organizations.

Another theoretical implication we find is the support of the proposed moderated mediation relationship. We find that when employees have higher supervisory support, it attenuates the harmful effects of relationship conflicts with co-workers on their validation of creative ideas through a positive team affective tone. Therefore, our research illustrates that employees who enjoy supervisory support can manage and regulate the negative effects of relationship conflicts. Past research has identified the positive impact of supervisory support on employee creativity (48) and innovation (49). Similarly, team effective tone has also been identified as an antecedent of creative idea validation (68, 73). Hence, there is a need to identify the underlying mechanism of how supervisory support results in creative idea validation by co-workers. Our research reveals the moderated mediation effects of supervisory support and positive team affective tone that helps to mitigate the harmful impact of relationship conflicts on creative idea validation by coworkers.

Finally, creativity researchers focused predominantly on investigating variance-focused creativity, whereas selection-focused creativity has received less attention (3). Therefore, creativity research needs to pay more attention to the receiving side of creativity (33). Thus, contemporary research on creativity demands an understanding of the factors that may hinder creative process due to interpersonal conflicts. This study also contributes to the receiving side of creativity research by investigating the detrimental effects of relationship conflicts for validation of creative ideas: the selection-focused receiving side of creativity process.

### Limitations and future research direction

Although we have made some valuable contributions to this research, this research should be considered light of limitations. The basic limitation of this study lies in the research design; the survey-based study makes it vulnerable to alternative explanations of the hypothesized relationships. A combination of correlation research and an experiment with different operationalization, controls, and manipulation brought a more precise and accurate picture of the causal inferences. Although we have strong theoretical reason to expect that relationship conflicts with co-workers would precede creative idea validation and not vice versa (22); causal inference can be explained in a better way in a combination of correlation study and an experiment.

Additionally, the conventional use of multiple data sources and dividing the data collection process into two points in time reduced the chances of common method biases; these two conservative steps also reduced sample size from 578 observations and 47 clusters to 243 observations and 41 clusters. The context of the study is also a potential limitation, as the data was collected from IT engineers of a software house; the employees in our sample were nested into different workgroups, which were distinguished based on their functional assignments. Therefore, we are unaware that the relationship among the study variables exists in other industries or from the sample with employees of different hierarchical levels. Thus, we recommend, further research should explore and operationalize the relationship among the variables in sectors other than information technology and the sample collected on multiple hierarchical levels.

## Data availability statement

The original contributions presented in the study are included in the article/supplementary material, further inquiries can be directed to the corresponding author.

## Ethics statement

The studies involving human participants were reviewed and approved by Dr. Zeeshan Ahmed. The patients/participants provided their written informed consent to participate in this study.

## Author contributions

All authors listed have made a substantial, direct, and intellectual contribution to the work and approved it for publication.

## Conflict of interest

The authors declare that the research was conducted in the absence of any commercial or financial relationships that could be construed as a potential conflict of interest.

## Publisher's note

All claims expressed in this article are solely those of the authors and do not necessarily represent those of their affiliated organizations, or those of the publisher, the editors and the reviewers. Any product that may be evaluated in this article, or claim that may be made by its manufacturer, is not guaranteed or endorsed by the publisher.
